# Efficacy of oral *Polypodium leucotomos*, colchicine cream and ingenol mebutate in the treatment of actinic keratoses and cutaneous field cancerization: a randomized clinical trial^☆^

**DOI:** 10.1016/j.abd.2022.04.006

**Published:** 2022-12-23

**Authors:** Anna Carolina Miola, Hélio Amante Miot

**Affiliations:** Department of Dermatology, Faculty of Medicine, Universidade Estadual Paulista, Botucatu, SP, Brazil

Dear Editor,

Cutaneous field cancerization (CFC) comprises an area of ​​clinically normal skin with genetically altered cells, justifying the high incidence of neoplasms, or the recurrence of completely excised tumors.^1^ Actinic keratosis (AK) is a manifestation of CFC the treatment of which may contribute to the reduction of skin tumors.^2^

*Polypodium leucotomos* (PL) contains polyphenols with possible antineoplastic activity. Treatment with photodynamic therapy (PDT) plus oral PL for six months reduced facial AK lesions by 88%, which was slightly higher than treatment with PDT alone (71%).^3^ Topical colchicine promoted a reduction in AK lesions similar to a session of PDT on the forearms.^4^ Ingenol mebutate (IM) provides 42% total clearance of AKs on the forearms, with a sustained effect after six months.^5^

To date, there are no studies comparing colchicine with IM in the treatment of CFC or evaluating the efficacy of oral PL.

A randomized, self-controlled, factorial, double-blind trial was carried out for oral treatment and an open-label trial for topical treatments, aiming to evaluate the effectiveness of 0.5% colchicine cream, 0.05% IM gel and oral PL in the treatment of AKs and CFC on the forearms.

The study was carried out at the Dermatology outpatient clinic of the UNESP Faculty of Medicine between May/2019 and December/2020. Fifty immunocompetent individuals with three to ten AKs on both forearms, with no other skin condition, were randomized to receive PL 500 mg orally 2×/day or a placebo tablet containing hydrocellulose at the same dose and in capsules of identical color and size (PLAC) for 60 days. After randomization of the oral treatment, their forearms were randomized to receive: Colchicine 0.5% cream 2×/day for 7 days (COL), IM 0.05% gel (Picato®, LEOPharma®) 1×/day for two days (IM), or just SPF30 sunscreen (Anthelios XL Protect®; SC). All patients were instructed to use sunscreen on their forearms. Thus, six groups of forearms undergoing treatment were formed: PL + COL, PL + IM, PL + SC, PLAC + COL, PLAC + IM and PLAC + SC.

The participants were assessed on D0 for inclusion, randomization, clinical assessment: AK count, actinic keratosis severity score (AKSS),^6^ Forearm photoaging scale (FPS)^7^ and the starting of treatment; on D15 for assessment of adverse events (AE); on D60 and D180 for further clinical evaluation.

Twelve patients were selected by convenience and biopsied on D0, D60, and D180, in the central region of each forearm, in an area free of lesions that were clinically compatible with AK, for evaluation of the KIN (keratinocyte intraepithelial neoplasia) score.^8^

The study was approved by the Ethics Committee, and the clinical trial was registered at REBEC (https://ensaiosclinicos.gov.br/rg/RBR-5q5dg8).

The primary endpoint was total AK clearance on D60. The secondary endpoints were: partial clearance (−50% AK) and reduction in CFC activity, analyzed by AKSS, FPS, and KIN, on D60 and D180, aiming at analyzing the maintenance of treatment response over time. The sample was sized to detect >20% reduction difference between the groups (power = 0.9; alpha = 0.05), resulting in 16 forearms per group (48 patients).

All participants included in the study were part of the ITT (Intention To Treat) population. The AK, AKSS, FPS and KIN evaluations were compared according to the time and groups using a generalized linear model of mixed effects. Missing values/dropouts were assigned by the mixed model analysis. The significance was set at a one-tailed p < 0.05.

There were 77 eligible patients for the study, but 27 were excluded (15 did not meet all the inclusion criteria on both forearms and 12 refused to participate). Fifty patients were then included so 25 were randomized to the PL group and 25 to the PLAC group. The groups were thus formed: PL + COL (17 forearms), PL + IM (16), PL + SC (17), PLAC + COL (17), PLAC + IM (15), and PLAC + SC (18). There were four dropouts, two on D60 and two on D180 (one for each oral treatment group), not associated with adverse effects of the treatments.

The main demographic characteristics of the participants are shown in Table 1. Total clearance on D60 was observed in two forearms (8.0%) in the PL group and three (12.0%) in the PLAC group, with no difference between them (p = 0.26). Regarding topical treatments, total clearance was obtained in five forearms (14.7%) in the COL group, five (16.1%) in the IM, and one (2.8%) in the SC group (p = 0.8). Partial clearance was achieved in 11 (44.0%) forearms in the PL group, 11 (44.0%) in the PLAC (p = 0.26); 18 (52.9%) in the COL, 17 (54.8%) in the IM and nine (25.7%) in the SC group (p = 0.24).

**Table 1:** Main demographic characteristics of the participants (n = 50).

Variables	PL (n = 25)	PLAC (n = 25)	Total
Age (years), mean (SD)	75 (7)	70 (8)	72 (8)
Sex, n (%)			
Male	17 (68)	15 (60)	32 (64)
Female	8 (32)	10 (40)	18 (36)
Phototype, n (%)			
I	3 (12)	4 (16)	7 (14)
II	18 (72)	14 (56)	32 (64)
III	4 (16)	7 (28)	11 (22)
Chronic exposure to sunlight, n (%)	24 (96)	24 (96)	48 (96)
Smoking, n (%)	7 (28)	9 (36)	16 (32)
Level of schooling, n (%)			
Elementary School	20 (80)	23 (92)	43 (86)
High school	4 (16)	2 (8)	6 (12)
Higher Education	1 (4)	- (-)	1 (2)
AK lesions, median (p25-p75)^a^	12 (11‒15)	11 (9‒12)	11 (10‒13)
FPS, median (p25-p75)^a^	208 (184‒212)	184 (144‒208)	194 (164‒212)
AKSS, median (p25-p75)^a^	20 (15‒25)	17 (13‒25)	18 (14‒25)

PL, *P. leucotomos* group; PLAC, Placebo group; FPS, Forearm Photoaging Scale; AKSS, Actinic keratosis severity score.

a Sum of the two treated forearms.

The results after 60 and 180 days for AK lesions, forearm photoaging scale, and AKSS are shown in Table 2 and Fig. 1.

**Table 2:** Main clinical outcomes resulting from treatments (n = 100 forearms).

Variable	PL + COL (n = 17)	PL + IM (n = 16)	PL + SC (n = 17)	PLAC + COL (n = 17)	PLAC + IM (n = 15)	PLAC + SC (n = 18)
AK						
D0	6.0 (1.7)	6.2 (1.5)	6.1 (1.6)	4.8 (1.5)	5.5 (1.2)	5.7 (1.4)
D60	**3.6 (3.0)**^a,c^	3.7 (2.2)^a^	**4.5 (2.1)**^a,c^	**2.6 (1.9)**^a,c^	4.5 (0.7)^a^	**4.1 (2.5)**^c^
D180	**2.6 (1.9)**^b,c^	3.0 (2.2)^b^	**4.4 (2.7)**^b,c^	**2.1 (1.8)**^b,c^	1.5 (2.2) ^b^	**2 (1.5)**^b,c^
FPS						
D0	94.8 (15.6)	95.8 (17.2)	94.3 (16.6)	88.8 (17.0)	88.5 (26.1)	91.1 (20.6)
D60	83.0 (15.0)^a^	82.9 (15.9)^a^	85.7(19.1)^a^	78.1 (23.0)^a^	74.5 (14.8)^a^	82.8 (20.1)
D180	**86.6 (13.9)**^b,c^	82.8 (16.7)^b^	**85.0(18.9)**^b,c^	**68.9 (18.0)**^b,c^	72.5 (26.1)^b^	**84.6 (12.8)**^c^
AKSS						
D0	10.2 (5.9)	10.6 (4.8)	9.7 (3.5)	8.2 (5.0)	10.5 (0.7)	8.6 (3.1)
D60	4.8 (4.1)^a^	5.5 (4.3)^a^	6.5 (3.7)^a^	3.4 (2.6)^a^	6.5 (3.5)	5.0 (3.4)
D180	**4.2 (4.4)^b,c,d^**	**6.1 (4.9)^b,d^**	**7.1 (6.3)^b,c,d^**	**3.5 (4.2)^b,c,d^**	**3.5 (4.95)^d^**	**3.3(2.4)^b,c,d^**

PL, *Polypodium leucotomos*; SC, sunscreen, SPF 30; IM, ingenol mebutate; COL, colchicine; AK, actinic keratosis counts; FPS, Forearm Photoaging Scale. AKSS, Actinic keratosis severity score.

^a^ p(T0 vs. T60) < 0.05; ^b^ p(T0 vs. T180) < 0.05; ^c^ p(SC vs. COL) < 0.05; ^d^ p(PL vs. PLAC) < 0.05.

Bold entries are significant results found for treatments prescribed.

**Figure 1:**
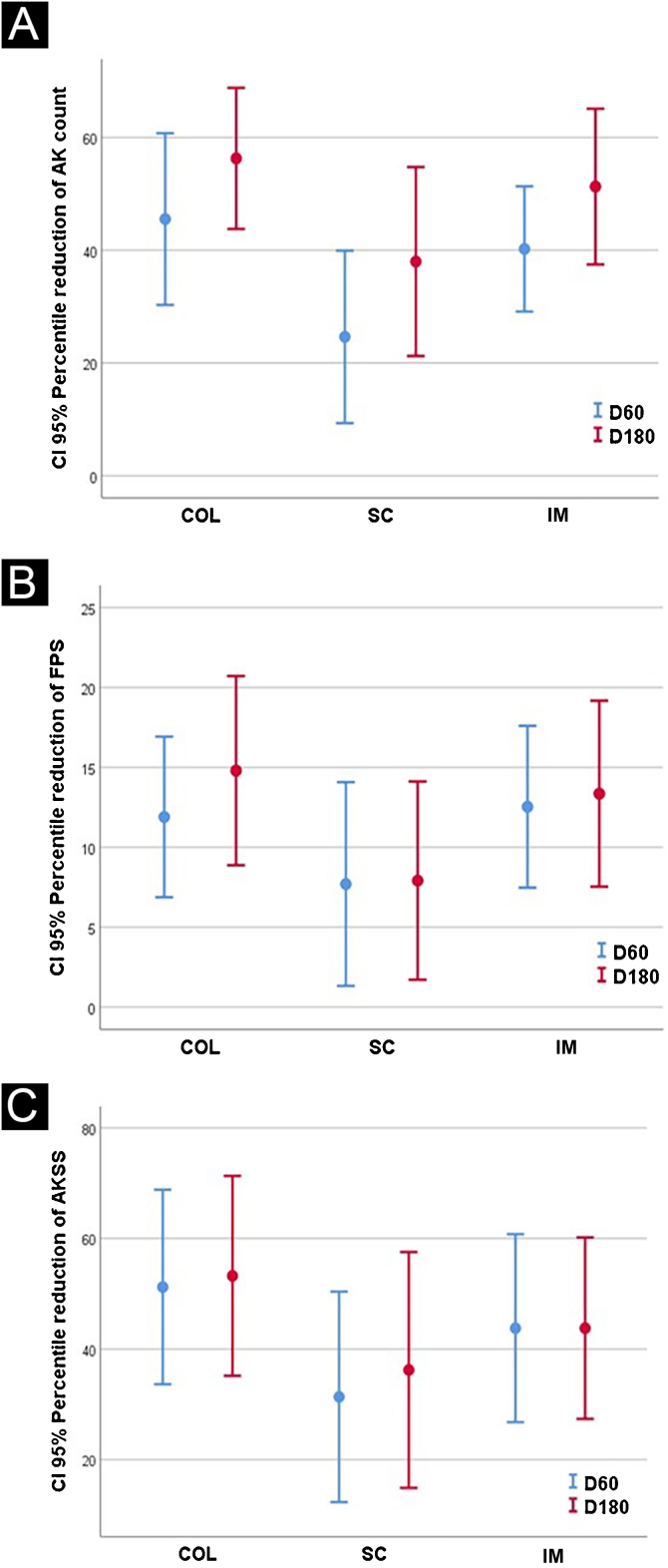
Confidence intervals: (A) AK count; (B) forearm photoaging scale (FPS) and (C) actinic keratosis severity score (AKSS).

The KIN analysis showed no interference of oral treatments on its behavior on D60 and D180, so the assessment was subsequently performed with topical treatments only. On the other hand, further analysis of the effect of topical treatments showed that COL reduced KIN after 60 and 180 days of treatment (p < 0.05), while the other treatments did not show a similar response (Table 3).

**Table 3:** Results of KIN for the groups COL, SC and IM (n = 24 forearms).

Variable	COL (n = 6)	SC (n = 8)	IM (n = 10)
D0			
KIN 0	0 (-)	0 (-)	0 (-)
1	1 (17%)	2 (25%)	2 (20%)
2	0 (-)	3 (37%)	7 (70%)
3	5 (83%)	3 (38%)	1 (10%)
D60			
KIN 0	1 (17%)	0 (-)	3 (30%)
1	0 (-)	2 (25%)	3 (30%)
2	4 (67%)	5 (62%)	3 (30%)
3	1 (17%)^a^	1 (13%)	1 (10%)
D180			
KIN 0	3 (50%)	0 (-)	2 (20%)
1	0 (-)	3 (37%)	3 (30%)
2	3 (50%)	3 (38%)	3 (30%)
3	0 (-)^b^	2 (25%)	2 (20%)

SC, SPF30 sunscreen; IM, ingenol mebutate; COL, colchicine.

^a^ p(T0 vs. T60) < 0.05; ^b^ p(T0 vs. T180) < 0.05.

As for the adverse events (AEs), two patients (8%) in the PLAC group had diarrhea and two in the PL group (8%) had epigastric pain. Regarding topical treatments, local moderate AEs were seen in 13% of the IM group, 23%, and 8% of the COL and SC groups.

The only trial with oral PL in CFC associated it with two sessions of PDT, with a greater reduction in scalp AK lesions when compared to PDT alone.^3^ Although the present study was performed on forearms, whose results are less significant, the previous study was not placebo-controlled, suggesting a possible bias.

Topical 0.5% colchicine cream twice a week for 10 days was compared with a session of PDT on forearms for treatment of AK, showing a total clearance of 17% and a reduction of 42% in AK lesions, with no differences in PDT.^4^ In the present study, total clearance was comparable (16%). Another observation is its superiority over SC even on D180 regarding AK lesions and FPS, suggesting a sustained response. Recently, tirbanibulin, a synthetic inhibitor of tubulin polymerization similar to colchicine, showed promising results in the treatment of AKs of the face, with total clearance in 54% of the patients evaluated two months after its topical use at 1% for five days in the treatment of CFC, which reinforces the positive evidence regarding the use of antiproliferative drugs.

IM has already been compared with some of the available treatments, showing similar efficacy to diclofenac and imiquimod, with the benefit of a shorter treatment regimen. On the other hand, the present study showed a lower clearance than that presented in the literature. In the present study, treated areas were larger than 25 cm^2^, as recommended by the manufacturer, which may have reduced the effectiveness of IM due to the lower concentration of the product per unit area.

The European Medicines Agency has suspended the marketing of IM after reviewing phase IV studies that showed a possible relationship with an increased risk of squamous cell carcinoma.^9^ One of the participants in the IM group developed, on D180, a keratoacanthoma on the forearm, emphasizing the importance of long-term follow-up. There is, therefore, the possibility of publication bias of studies with IM in the treatment of AK, explaining the controversy between the results.

The loss of nuclear polarization contributes to the characterization of CFC activity.^10^ The reduction of KIN by colchicine is evidence of its effects on keratinocyte proliferation. This same parameter was evaluated in a previous study, with similar results after 60 days of treatment.^4^ This study is the first to assess the sustained response of keratinocyte dysplasia after colchicine use. More studies are needed, including different doses and routes rather than topical colchicine, aiming to assess the histopathological reduction of CFC activity.

This study has limitations: it was conducted in a single center in the Brazilian Unified Health System (SUS, *Sistema Único de Saúde*), which minimizes the generalization of the results. Additionally, both PL and colchicine were produced in a manipulation pharmacy, which may result in some variability in relation to the industrial product.

In conclusion, colchicine showed to be effective and tolerable in the treatment of AKs and CFC, also improving the signs of photodamage on the forearms, which may represent an alternative in the treatment of CFC, especially for the low-income population.

## Authors' contributions

Miola AC: Design and planning of the study; effective participation in research orientation; collection, analysis, and interpretation of data; critical review of the literature; critical review of the manuscript; writing and approval of the final version of the manuscript.

Miot HA: Design and planning of the study; effective participation in research orientation; project development, analysis and interpretation of data; critical review of the literature; critical review of the manuscript, writing and approval of the final version of the manuscript.

## Financial support

FUNADERM (Fundo de Apoio à Dermatologia).

## Conflicts of interest

None declared.

## References

[1] Curtius K., Wright N.A., Graham T.A. (2017). An evolutionary perspective on field cancerization. Nat Rev Cancer..

[2] Reinehr C.P.H., Bakos R.M. (2019). Actinic keratoses: review of clinical, dermoscopic, and therapeutic aspects. An Bras Dermatol..

[3] Auriemma M., Di Nicola M., Gonzalez S., Piaserico S., Capo A., Amerio P. (2015). Polypodium leucotomus supplementation in the treatment of scalp actinic keratosis: could it improve the efficacy of photodynamic therapy?. Dermatol Surg..

[4] Miola A.C., Ferreira E.R., Lima T.R.R., Schmitt J.V., Abbade L.P.F., Miot H.A. (2018). Effectiveness and safety of 0·5% colchicine cream vs. photodynamic therapy with methyl aminolaevulinate in the treatment of actinic keratosis and skin field cancerization of the forearms: a randomized controlled trial. Br J Dermatol..

[5] Hanke C.W., Albrecht L., Skov T., Larsson T., Østerdal M.L., Spelman L. (2020). Efficacy and safety of ingenol mebutate gel in field treatment of actinic keratosis on full face, balding scalp, or approximately 250 cm^2^ on the chest: a phase 3 randomized controlled trial. J Am Acad Dermatol..

[6] Arruda G.O., Miola A.C., Miot H.A., Schmitt J.V. (2022). Clinical characteristics of actinic keratoses and their histological correlations: suggestion for a clinical severity scale. Surg Cosmet Dermatol..

[7] Guimarães C.O.Z., Bagatin E., Guadanhim L.R.S., Sternberg F., Picosse F.R., Nunes G. (2015). Development and validation of a clinical scale for the evaluation of forearm skinphotoaging. J Cutan Med Surg..

[8] Anwar J., Wrone D.A., Kimyai-Assadi A., Alam M. (2004). The development of actinic keratosis into invasive squamous cell carcinoma: evidence and evolving classification schemes. Clin Dermatol..

[9] US National Library of Medicine. Risk of squamous cell carcinoma on skin areas treated with ingenol mebutate gel, 0.015% and imiquimod cream, 5%. [cited 2022 Mar 08]. Available from: https://clinicaltrials.gov/ct2/show/NCT01926496.

[10] Miola A.C., Castilho M.A., Schmitt J.V., Marques M.E.A., Miot H.A. (2019). Contribution to characterization of skin field cancerization activity: morphometric, chromatin texture, proliferation, and apoptosis aspects. An Bras Dermatol..

